# Essential amino acid ratios and mTOR affect lipogenic gene networks and miRNA expression in bovine mammary epithelial cells

**DOI:** 10.1186/s40104-016-0104-x

**Published:** 2016-08-03

**Authors:** Shanshan Li, Afshin Hosseini, Marina Danes, Carolina Jacometo, Jianxin Liu, Juan J. Loor

**Affiliations:** 1 Institute of Dairy Science, College of Animal Sciences, Zhejiang University, Yuhangtang Road 866, Hangzhou, 310058 People’s Republic of China; 2 Department of Animal Sciences, Mammalian NutriPhysioGenomics, University of Illinois, 1207 West Gregory Drive, Urbana, IL 61801 USA; 3 Department of Animal Science, University of Lavras, Lavras, MG 37200-000 Brazil; 4 NUPEEC, Departamento de Clínicas Veterinária, Programa de Pós-Graduação em Biotecnologia, Universidade Federal de Pelotas, 96010-900 Pelotas, RS Brazil

**Keywords:** Amino acids, Milk fat synthesis, mTOR, Nutrigenomics

## Abstract

**Background:**

The objective of this study was to study how changing the ratio of Lys to Thr, Lys to His, and Lys to Val affects the expression of lipogenic genes and microRNA (miRNA) in bovine mammary epithelial cells.

**Results:**

Triplicate cultures with the respective “optimal” amino acid (AA) ratio (OPAA = Lys:Met 2.9:1; Thr:Phe 1.05:1; Lys:Thr 1.8:1; Lys:His 2.38:1; Lys:Val 1.23:1) plus rapamycin (OPAARMC; positive control), OPAA, Lys:Thr 2.1:1 (LT2.1), Lys:Thr 1.3:1 (LT1.3), Lys:His 3.05:1 (LH3.0), or Lys:Val 1.62:1 (LV1.6) were incubated in lactogenic medium for 12 h. The expression of 15 lipogenic genes and 7 miRNA were evaluated. Responses to LT2.1, LT1.3, LH3.0, and LV1.6 relative to the control (OPAARMC) included up-regulated expression of *ACSS2, FABP3, ACACA, FASN, SCD, LPIN1, INSIG1, SREBF1, PPARD,* and *NR1H3* (commonly known as *LXR-α*). Furthermore, LV1.6 up-regulated expression of *ACSL1, DGAT1*, and *RXRA* and down-regulated *PPARG* expression. Although no effect of OPAA on expression of *PPARG* was observed, compared with the control, OPAA up-regulated expression of the PPAR targets *ACSS2, FABP3, ACACA, FASN, SCD, LPIN1, INSIG1,* and *SREBF1*. Compared with the control, the expression of the anti-lipogenic MIR27AB was down-regulated by OPAA, LT2.1, LT1.3 and LH3.0. In contrast, compared with the control, the expression of the pro-lipogenic MIR21 was up-regulated by LT2.1, LT1.3, LH3.0, and LV1.6.

**Conclusions:**

The observed up-regulation of lipogenic gene networks and the changes in expression of key miRNA involved in the control of lipogenic balance are indicative of a potentially important role of EAA ratios and mTOR signaling in the regulation of milk fat synthesis.

**Electronic supplementary material:**

The online version of this article (doi:10.1186/s40104-016-0104-x) contains supplementary material, which is available to authorized users.

## Background

Previous research has underscored that provision of adequate levels of essential AA (EAA) is critical for improving N utilization efficiency as well as maximizing bovine milk protein synthesis [1]. Rulquin et al. [2] proposed “ideal” values for intestinal absorption of EAA in dairy cows; however, after absorption, AA flow first to the liver where substantial and differential net removal occurs leading to marked alterations in the pattern of AA that can be supplied to the mammary gland [3]. Hence, studies evaluating the effects of EAA in mammary cells are essential to improve our understanding of their metabolism and the underlying mechanisms [4–7]. Such experiments have been instrumental in efforts to improve the efficiency of dietary protein use in dairy cows by allowing the development of mechanistic models that can help guide the design of future experiments [8].

Both Lys and Met, at supraphysiological concentrations, are known to stimulate β-casein synthesis in dairy cow mammary epithelial cells (MEC) [5, 6]. It was demonstrated in murine MEC that Lys, His, and Thr when added to an AA-depleted medium, exerted negative effects on S6K1 and 4E-BP1 phosphorylation via the specific AA pathway that signals to mTOR complex 1 (mTORC1) [9]. Subsequent studies using bovine MEC have explored the effects of individual EAA on milk protein mRNA expression, cell signaling and milk protein synthesis [5–7]. However, an unexplored area of EAA metabolism within bovine MEC is the potential effect of these nutrients on gene expression of lipogenic target genes. The fact that mTORC1 is required for Akt-mediated induction of SREBP-1c in retinal pigment epithelial cells [10] indicates a potential link between AA and lipogenesis. Indeed, a putative role for AA on lipogenesis has been uncovered using non-ruminant hepatocytes where Met and Lys plus insulin not only altered mTOR signaling but also the expression of fatty acid (FA) synthase (*FASN*) [11]. Furthermore, Leu and Gln increased activity of acetyl-CoA carboxylase (*ACACA*) [12] in rat hepatocytes.

A “missing link” between EAA availability and alterations in lipogenic gene expression in the studies reported to date could be alterations in peroxisome proliferator-activated receptor-γ (*PPARG*) and/or sterol-responsive element binding factor 1 (*SREBF1*) expression and/or signaling. Both of these transcription regulators are up-regulated (along with their target genes) in bovine mammary gland at the onset and throughout lactation [13]. Evidence for the control of *PPARG* via microRNA (miRNA) was reported recently in studies where up-regulation of MIR27AB led to downregulation of *PPARG* expression and lipogenesis in adipocytes [14]. Hence, this miRNA and others such as MIR34A, MIR130A, and MIR448 [15] have the potential to control mammary lipogenesis through negative effects on PPARG. In the context of controlling the cellular lipogenic balance, it is noteworthy that *PPARG* itself alters transcription of MIR103 and MIR378, hence, these miRNA could elicit a pro-lipogenic effect through targets such as *FASN* [16]. Recent work also provides evidence for a positive cooperative mechanism between MIR21 and SREBF1 in the control of lipogenesis [17].

The general hypothesis was that the EAA profile could affect mRNA expression of lipogenic gene networks and selected lipogenic miRNA in bovine mammary cells. Because of the newly-demonstrated linkage between mTOR signaling and activation of lipogenesis via the Liver-X-receptor α (gene symbol *NR1H3*) and SREBF1 [15], it was important to determine gene transcription relative to the inhibition of mTOR signaling using Rapamycin. Therefore, the specific objective was to culture MEC with specific EAA ratios and profile the mRNA expression of several lipogenic genes as well as lipogenic/anti-lipogenic miRNA.

## Methods

### Cell culture and treatments

Bovine mammary epithelial cells (Mac-T) were allowed to grow in canted neck, vented, 75 cm^2^ flasks (430641, Corning, Glendale, AZ) in Minimum Essential Medium/Earle’s Balanced Salts HyQ (MEM/EBSS, HyClone, Logan, UT). Cultures were maintained in a water-jacketed incubator (model 3158, Forma Scientific, Marietta, OH) with 5 % in air at 37 °C. Culture medium was changed every 24 h and cells were subcultured to 80 to 90 % confluence by rinsing once with (10 mL) of PBS buffer without Ca and Mg (sh3002802, HyClone), 3 mL of 0.25 % trypsin (sh3004201, HyClone) plus 3 mL of Cellstripper (25-056-Cl, Cellgro, Herndon, VA), and incubated at 37 °C for 5 to 10 min (i.e., until evidence of cell detachment). Trypsin activity was inhibited by addition of 6 mL of fresh culture media at 37 °C.

Approximately 48 h prior to the last subculture before initializing the experiment, cells were allowed to grow in a basal medium composed of Minimum Essential Medium/Earle’s Balanced Salts HyQ (MEM/EBSS, HyClone), insulin (5 mg/L, NC0374447, Fisher Scientific, Pittsburgh, PA), hydrocortisone (1 mg/L, H0888, Sigma, St. Louis, MO), transferrin (5 mg/L, T1428, Sigma), ascorbic acid (5 μmol/L, A4544, Sigma), sodium acetate (5 mmol/L, S5636, Sigma), and penicillin/streptomycin (10 mL/L, P4333, Sigma). The basal medium was supplemented with fetal bovine serum (10 %, FBS, SH3007002, HyClone) and growth-promoting hormones (1 mg/L of progesterone, P8783, Sigma; 0.05 % lactalbumin, L5385, Sigma; and 0.05 % α-lactose, 47287-U, Sigma). When the cells reached ~90 % confluence they were cultured overnight with 2.5 mL of a lactogenic medium in 6-well plates as reported by Kadegowda et al. [18]. The lactogenic medium was prepared as the basal medium, except that essential AA-free high-glucose Dulbecco’s modified Eagle’s medium (HG-DMEM, Gibco Custom Media, Invitrogen) was used. The AA composition of the basal medium is reported in Table 1. The lactogenic medium was devoid of fetal bovine serum and was supplemented with BSA (1 g/L), prolactin (2.5 mg/L) and EAA to achieve the different AA treatment ratios using the required amount of each individual EAA (L-isomer; Sigma-Aldrich) to the lactogenic medium.

**Table 1:** Amino acid composition of experimental treatments

	Treatments^a^					
	OPAARMC	OPAA	LT1.3	LT2.1	LH3.0	LV1.6
Experiment, μg/mL						
Lys	175	175	175	175	175	175
Met	60	60	60	60	60	60
Lys/Met	2.9:1	2.9:1	2.9:1	2.9:1	2.9:1	2.9:1
Thr	97	97	135	83	97	97
Phe	92	92	128	79	92	92
Thr/Phe	1.05:1	1.05:1	1.05:1	1.05:1	1.05:1	1.05:1
Lys/Thr	1.8:1	1.8:1	1.3:1	2.1:1	1.8:1	1.8:1
His	74	74	74	74	57	74
Lys/His	2.38:1	2.38:1	2.38:1	2.38:1	3.05:1^b^	2.38:1
Val	142	142	142	142	142	108
Lys/Val	1.23:1	1.23:1	1.23:1	1.23:1	1.23:1	1.62:1^c^
Rapamycin, ng/mL	9.147	-	-	-	-	-
Other amino acids, μg/mL						
Arg	84	84	84	84	84	84
Cys	63	63	63	63	63	63
Gln	584	584	584	584	584	584
Gly	30	30	30	30	30	30
Ile	105	105	105	105	105	105
Leu	105	105	105	105	105	105
Ser	42	42	42	42	42	42
Trp	16	16	16	16	16	16
Tyr	104	104	104	104	104	104

^a^OPAARMC = optimal amino acid ratios with rapamycin (control); OPAA = optimal amino acid; LT2.1 = Lys:Thr at 2.1; LT1.3 = Lys:Thr at 1.3; LH3.0 = Lys:His at 3.05; LV1.6 = Lys:Val at 1.62. Amino acid ratios derived from Rulquin et al. [2]. Experimental treatments were designed by adding the required amount of the essential AA to the basal lactogenic essential AA-free media (HG-DMEM, Custom Media, Invitrogen) to reach the desired concentration

^b^From Lee et al. [19]

^c^From Haque et al. [22]

Triplicate cultures were incubated for 12 h with the following treatments (Table 1): “optimal” AA ratio (OPAA = Lys:Met 2.9:1; Thr:Phe 1.05:1; Lys:Thr 1.8:1; Lys:His 2.38:1; Lys:Val 1.23:1), OPAA plus Rapamycin (OPAARMC, control), Lys:Thr 2.1:1 (LT2.1), Lys:Thr 1.3:1 (LT1.3), Lys:His 3.05:1 (LH3.0), and Lys:Val 1.62:1 (LV1.6). The OPAA was designed to resemble the profiles proposed by Rulquin et al. [2]; the other treatments were designed to change the specific ratio described in the treatments by keeping Lys constant and altering the concentration of the other AA, while also keeping the other AA constant as in the OPAA. Compared with OPAA, LT1.3 was 39 % greater in Thr and Phe, LT2.1 was 14 % lower in Thr and Phe, LH3.0 was 23 % lower in His, and LV1.6 was 24 % lower in Val. The concentrations of AA in the experimental media were approximately 8 times the normal physiological concentrations in plasma, ranging across EAA from 4 to 20 times the normal concentration. The decision to increase the ratio of Lys to His was based on the observation that removal of rumen-protected His in a diet predicted to be limiting in MP resulted in lower milk protein and fat yields compared with a diet containing rumen-protected Lys, Met, and His [19]. In that study, addition of His increased milk fat yield by 30 g/d compared with the MP-deficient diet. Despite the lack of effect on milk fat yield, the higher ratio of Lys to Val used in the present study was to mimic the lowest level of Val recommended in the literature [20]. Similarly, the chosen ratio of Thr to Phe was to maintain it as close as possible to the theoretical optimal (~1.0:1.0) as discussed in Haque et al. [1]. The Lys to Thr ratios were chosen based on data from Prizant and Barash [9].

### Preliminary time-course study

The Mac-T cells from several 75 cm^2^ flasks were pooled after trypsinization in a 50-mL tube and mixed thoroughly before transferring to 6-well plates for addition of treatments. As reported above, cultures were maintained for 24 h in a lactogenic medium before treatments were applied (~90 % confluence). For RNA extraction, cells were cultured in duplicate to generate three specific treatments: an essential AA-free treatment (−EAA, HG-DMEM without EAA), OPAARMC and OPAA. These treatments were performed for 0, 1, 6, 12 h. Cells were harvested in 1 mL of QIAzol reagent (Invitrogen) and stored at −80 °C until RNA extraction.

The preliminary study was conducted to determine a suitable incubation time based on peak mRNA expression of the genes eukaryotic translation initiation factor 4E, eukaryotic translation initiation factor 4E-binding protein 1, mechanistic target of rapamycin (Ser/Thr kinase), ras homolog enriched in brain, ribosomal protein S6 kinase beta-1, and tuberous sclerosis 1 (data not shown). The final data for statistical analysis were calculated as the percentage change relative to time 0 for all treatments. Statistical analysis was performed using the MIXED procedure of SAS (version 9.1, SAS Institute, Cary, NC) to evaluate the effects of treatment, time, and treatment × time on normalized mRNA expression of genes. The model included the fixed effect of time (0, 1, 6, and 12 h), treatment (−EAA, OPAARMC, and OPAA), and the random effect of replicate nested within treatment and time. Results (not shown) indicated that a 12-h incubation was suitable to obtain maximal responses in mRNA expression. Clearly, this represented a compromise because it is unlikely that all genes would have maximal expression at the same time point.

### Main experiment

To obtain a large number of cells to initiate the main experiment, several subcultures were performed. When the numbers of cells were deemed adequate to initiate the experiment, all the cells were pooled in a 50-mL sterile tube with fresh medium at 37 °C and mixed thoroughly before plating in 75 cm^2^ flasks with lactogenic medium. In doing this we attempted to obtain the same initial number of cells in each flask and, thus, ensure consistency for the initial conditions among all samples. Before initiating the experiment, cells remained in the basal medium (changed every 24 h) for approximately 2 d, when they reached ≥ 90 % confluence. Subsequently, cells were cultured in lactogenic medium overnight before the treatments were applied. The OPAA, OPAARMC, LT2.1, LT1.3, LH3.0, and LV1.6 treatments in lactogenic medium (as described above in the “cell culture and treatments” section were incubated in triplicate (i.e., 3 separate flasks per treatment) before the cells were harvested after 12 h of incubation.

### RNA extraction and quantification

Total RNA was extracted from the cells using the RNeasy Mini Kit columns (Qiagen, Valencia, CA). Concentrations of RNA were measured with a NanoDrop ND-1000 spectrophotometer (NanoDrop Technologies, Wilmington, DE). RNA integrity (RIN) was assessed using Aglient 2100 Bioanalyzer (Aglient Technologies, Senta Clara, CA). The RIN factor was above 7.5. A portion of the RNA was diluted to 100 ng/μL using RNase free water before reverse transcriptase. Adequate cDNA was prepared to run all selected genes. Each cDNA was synthesized using 100 ng RNA, 1 μL Random Primers (Roche, Basel, Switzerland), and 9 μL RNase free water. The mixture was incubated at 65 °C for 5 min in an Eppendorf Mastercycler Gradient and keep on ice for 3 min. A total of 9 μL of Master Mix, composed of 4 μL 5 × First-Strand Buffer (Fermentas, Pittsburgh), 1 μL Oligo dT18, 2 μL 10 mmol/L deoxynucleotide 5′-triphosphate mix (Invitrogen), 0.25 μL Reverse aid RT (Fermentas), 0.125 μL RNase Inhibitor (Fermentas), and 1.625 μL RNase free water was added. The reaction was performed using the following temperature program: 25 °C for 5 min, 42 °C for 60 min, and 70 °C for 5 min. Complementary DNA was then diluted 1:4 with RNase free water.

The qPCR was performed in a MicroAmp Optical 384-well reaction plate (Applied Biosystems, Foster City, CA) using 4 μL diluted cDNA combined with 6 μL of a mixture composed by 5 μL 1 × SYBR Green Master Mix (Quanta, Gaithersburg, MD), 0.4 μL each of 10 μmol/L forward and reverse primers, and 0.2 μL RNase free water. Each sample was run in triplicate and a 6-point relative standard curve plus the nontemplate control were used. The reactions were performed in an ABI Prism 7900 HT SDS instrument (Applied Biosystems) under the following conditions: 5 min at 95 °C, 40 cycles of 1 s at 95 °C, and 30 s at 60 °C. The presence of a single PCR product was verified by the dissociation protocol using incremental temperatures to 95 °C for 15 s, 65 °C for 15 s, and 95 °C for 15 s. Data were analyzed using with the 7900 HT Sequence Detection Systems Software (version 2.4, Applied Biosystems). Methods for primer design and validation were reported previously [18]. Additional details on selected genes and real-time PCR performance for each gene analyzed are reported in Additional file 1: Table S1.

### Calculation and statistical analysis

The relative percentage of mRNA abundance among genes was analyzed as reported previously [13]. The qPCR data were normalized using the geometric mean of the three internal control genes (*GADPH, UXT*, and *RPS9*) [21]. The MIXED procedure of SAS (version 9.1, SAS Institute, Cary, NC) was used to evaluate the treatment effects on mRNA expression (fold-change) relative to the control (OPAARMC). Fixed effects in the model were treatments, whereas the random effect was replicate nested within treatment (*n* = 3 cultures/treatment). The Kenward-Roger statement was used for computing the denominator degrees of freedom. Treatment means were compared using the PDIFF statement in SAS after correction using Tukey’s. Significance was declared at a PDIFF Tukey-adjusted *P* < 0.05.

## Results

### Genes associated with FA metabolism

**Fig. 1:**
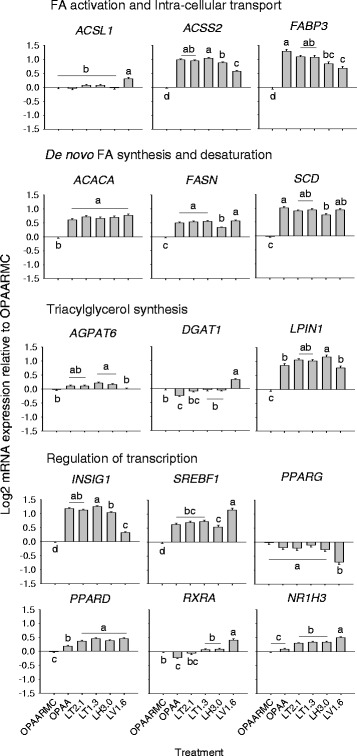
Expression of genes involved in fatty acid (FA) activation and intracellular FA transport, *de novo* FA synthesis and FA desaturation, triacylglycerol synthesis, and regulation of transcription. Superscript letters denote significant differences among treatments (*P* < 0.05). OPAARMC = optimal amino acid with rapamycin (control); OPAA = optimal amino acid; LT2.1 = Lys:Thr at 2.1; LT1.3 = Lys:Thr at 1.3; LH3.0 = Lys:His at 3.05; LV1.6 = Lys:Val at 1.62. *ACSS2* = acyl-CoA synthetase short-chain family member 2; *ACSL1* = acyl-CoA synthetase long-chain family member 1; *FABP3* = FA-binding protein, heart; *ACACA* = acetyl-coenzyme A carboxylase alpha; *FASN* = FA synthase; *SCD* = stearoyl-CoA desaturase; *AGPAT6* = 1-acylglycerol-3-phosphate O-acyltransferase 6; *DGAT1* = diacylglycerol acyltransferase 1; *LPIN1* = lipin 1; *INSIG1* = insulin induced gene 1; *SREBF1* = sterol regulatory element-binding transcription factor 1; *PPARG* = peroxisome proliferator-activated receptor gamma; *PPARD* = peroxisome proliferator-activated receptor beta; *NR1H3* = liver X receptor α; and *RXRA* = retinoid X receptor alpha

The responses of genes associated to FA metabolism are reported in Fig. 1. The mRNA expression of acyl-CoA synthetase long-chain family member 1 (*ACSL1*) was greatest with LV1.6 (*P* < 0.01) compared with other treatments. Compared with the control (OPAARMC), the expression of acyl-CoA synthetase short-chain family member 2 (*ACSS2*) and fatty acid-binding protein 3, muscle and heart (*FABP3*) was greater with OPAA, LT2.1, LT1.3, LH3.0 and LV1.6. Incubation with LT1.3 up-regulated the expression of *ACSS2* compared with LH3.0 (*P* < 0.01) and LV1.6 (*P* < 0.01), while LV1.6 down-regulated *ACSS2* compared with LH3.0 (*P* < 0.01). Treatment with OPAA led to greater expression of *FABP3* compared with LH3.0 (*P* = 0.04) and LV1.6 (*P* < 0.01), while incubation with LV1.6 resulted in lower expression compared with LT2.1 (*P* < 0.01) and LT1.3 (*P* < 0.01).

Among the genes associated with de novo FA synthesis and desaturation, acetyl-coenzyme A carboxylase alpha (*ACACA*), fatty acid synthase (*FASN*) and stearoyl-CoA desaturase (*SCD*) had greater expression in response to OPAA, LT2.1, LT1.3, LH3.0 and LV1.6 compared with the control. The LH3.0 resulted in lower expression of *FASN* compared with the other treatments (*P* < 0.01), while the expression of *SCD* was up-regulated in OPAA compared with LH3.0 (*P* < 0.01).

Expression of 1-acylglycerol-3-phosphate O-acyltransferase 6 (*AGPAT6*) was up-regulated with LT1.3 (*P* < 0.01) and LH3.0 (*P* < 0.01) compared with the control. The mRNA expression of diacylglycerol acyltransferase 1 (*DGAT1*) was down-regulated with OPAA compared with the control (*P* < 0.01), but was upregulated by LV1.6 compared with the other treatments (*P* = 0.01). The mRNA abundance of lipin 1 (*LPIN1*) was greater (*P* = 0.04) with OPAA, LT2.1, LT1.3, LH3.0 and LV1.6 compared with the control, while LH3.0 had greater (*P* < 0.01) expression compared with OPAA and LV1.6.

### Genes associated with regulation of transcription

The responses of genes associated with regulation of transcription are reported in Fig. 1. The expression of insulin induced gene 1 (*INSIG1*) and sterol regulatory element-binding transcription factor 1 (*SREBF1*) was up-regulated (*P* < 0.05) with OPAA, LT2.1, LT1.3, LH3.0 and LV1.6 compared with the control. Furthermore, incubation with LT1.3 up-regulated the expression of *INSIG1* compared with LH3.0 (*P* = 0.02) and LV1.6 (*P* < 0.01), while LV1.6 resulted in lower expression compared with LH3.0 (*P* < 0.01). For SREBF1, treatment with LV1.6 resulted in greater expression compared with the other treatments (*P* < 0.01). The mRNA expression of peroxisome proliferator-activated receptor gamma (*PPARG*) was lowest with LV1.6 compared with other treatments (*P* < 0.01). Compared with the control, expression of peroxisome proliferator-activated receptor beta (*PPARD*) was up-regulated with OPAA, LT2.1, LT1.3, LH3.0 and LV1.6 (*P* < 0.01). In contrast, OPAA resulted in lower expression of LT2.1, LT1.3, LH3.0 and LV1.6 (*P* < 0.01). The expression of retinoid X receptor alpha (*RXRA*) was down-regulated with OPAA (*P* = 0.01) and up-regulated with LV1.6 (*P* < 0.01) compared with the control. The expression of liver X receptor α (*NR1H3*) was up-regulated (*P* < 0.01) with LT2.1, LT1.3, LH3.0 and LV1.6 compared with the control, while LV1.6 resulted in greater (*P* < 0.01) expression than LT2.1, LT1.3 and LH3.0.

### MicroRNA expression

**Fig. 2:**
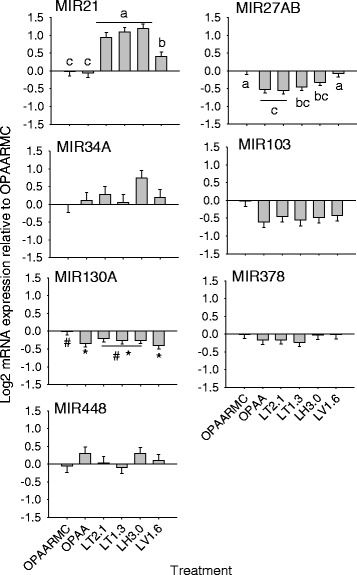
Expression of MIR21, MIR27AB, MIR34A, MIR103, MIR130A, MIR378 and MIR448. Superscript letters denote significant differences among treatments (*P* < 0.05). Symbols denote a tendency (*P* = 0.07) for significant differences among treatments. OPAARMC = optimal amino acid with rapamycin (control); OPAA = optimal amino acid; LT2.1 = Lys:Thr at 2.1; LT1.3 = Lys:Thr at 1.3; LH3.0 = Lys:His at 3.05; LV1.6 = Lys:Val at 1.62

MicroRNA expression responses to treatments are reported in Fig. 2. Unlike the lipogenic target genes, only 2 of the 7 miRNA evaluated were affected by the treatments (Fig. 2). Compared with OPAA and the control, the expression of MIR21 was greater (*P* < 0.01) in response to LT2.1, LT1.3, LH3.0 and LV1.6. Treatment with LV1.6 resulted in lower (*P* < 0.01) expression of MIR21 compared with LT2.1, LT1.3 and LH3.0. Compared with the control, the expression of MIR27AB was lower (*P* < 0.01) in response to OPAA, LT2.1, LT1.3 and LH3.0, while LV1.6 led to similar expression than the control.

## Discussion

### Indirect regulation of mammary lipogenic gene networks via the mTOR pathway

Signaling through mTOR plays a crucial role in mammary protein synthesis, and some amino acids, e.g. Leu, Ile and Val, are known to be potent stimulators of mTOR signaling and protein synthesis in mouse and bovine [5–7]. Other EAA such as Lys, His and Thr inhibited the mTOR pathway in mammary cells when added at supraphysiological concentrations to AA-depleted cell culture medium [9]. The fact that some EAA are potent activators of the mTOR signaling pathway and alter lipogenic gene transcription in non-ruminants [11, 12] led us to infer that a similar mechanism could exist in bovine mammary cells. In our study, the addition of rapamycin was employed to block mTOR signaling and differentiate the effects of AA ratios from mTOR signaling. A putative negative-feedback inhibition of Akt by mTOR was ruled out because previous research with mammary cells utilizing a complete AA mixture revealed no change in phosphorylation status of Akt after the addition of rapamycin [9].

Because the OPAA treatment stimulated gene expression of enzymes related to *de novo* FA synthesis (*ACACA, FASN*) and desaturation (*SCD*), triacylglycerol (TAG) synthesis (*LPIN1*), and transcription regulation of lipid synthesis (*SREBF1, INSIG1*), relative to OPAARMC the present data demonstrated that mTOR is, in fact, a regulator of lipid metabolism in mammary cells. The role of mTOR in lipogenesis has been previously demonstrated in murine hepatic cells [22, 23]. In fact, mTOR was essential for insulin-induced hepatic lipogenesis through the IRS-P13K-Akt pathway [23]. The mechanism proposed by these authors for mTOR regulation of lipogenesis is related to Akt-mediated phosphorylation of mTOR increasing gene expression of *SREBF1* in an S6K-independent manner [23]. The stimulation of *SREBF1* expression when OPAA was added to the medium without rapamycin, relative to OPAARMC, is further suggestive of this mechanism.

Evidence indicates that *SREBF1* plays an important role regulating lipid synthesis in bovine mammary epithelial cells [24]. Ma and Corl [24] reported that the expression of *ACSS2, FABP3, SCD*, and *LPIN1* decreased by blocking transcription of *SREBF1* with small interfering RNA technology. The response was indicative of a positive correlation among these genes. Therefore, the up-regulation of *SREBF1* with OPAA may have contributed to the greater expression of genes related to de novo FA and TAG synthesis.

Regarding the pathway by which mTOR stimulates *SREBF1*, Peterson et al. [25] demonstrated that mTORC1 phosphorylates lipin (LPIN1) in a murine hepatic cell line, which prevents its nuclear entry and subsequent inhibition of SREBF1. However, lipin plays a dual role in hepatic cells, both as transcriptional co-activator and as an enzyme in TAG synthesis [26]. Therefore, the *LPIN1* gene expression measured in this experiment is more likely reflective of its enzymatic role, which is in agreement with the upregulation of the FA synthesis enzymes. Because we did not attempt to determine intracellular location of lipin protein, we cannot infer directly about its function as transcriptional co-activator.

Another pathway that links mTOR with lipogenic regulation is through S6K-mediated phosphorylation of liver-X-receptor-α (*NR1H3*), a transcriptional regulator that senses oxysterols, dimerizes with retinoid-X-receptor-α (*RXRA*) and activates transcription of *SREBF1*, *FASN*, *SCD* and *ACACA* [27]. Liver-X-receptor-α (*NR1H3*) is a potentially-important transcription regulator of milk fat synthesis that enhances de novo FA synthesis in bovine MEC treated with the NR1H3 agonist T0901317 [28]. Despite the fact that no effect of rapamycin was observed on NR1H3, it is still possible that mTORC1 activation stimulated phosphorylation of the NR1H3 product, liver-X-receptor-α. However, the downregulation of *RXRA* expression with OPAA relative to OPAARMC is a stronger indication that NR1H3 was not involved in the observed lipogenic up-regulation, because *NR1H3* needs to form heterodimers with *RXRA* to regulate the transcription of lipogenic enzymes [29]. Therefore, despite evidence in non-ruminant hepatocytes of an LXR-mediated mTOR lipogenic effect, additional studies will have to be performed to better understand the mechanisms whereby NR1H3 may contribute to the regulation of lipogenic genes in bovine mammary gland.

In the absence of up-regulation of *NR1H3* with OPAA, the increase in mRNA expression of genes related to de novo synthesis (*ACACA* and *FASN*) and desaturation (*SCD*) might have been controlled by other transcriptional regulators such as *SREBF1* and *INSIG1*. This idea is supported by data from Oppi-Williams et al. [30], who reported lower *SCD* mRNA expression in *SREBF1*-knockdown Mac-T cells [30]. However, the higher expression of *INSIG1*, if extended to the protein level, might have resulted in a lower activity of SREBF1, which would have been retained in the ER via SCAP-INSIG1 binding [31], despite its concomitant higher expression.

This would suggest the involvement of other key transcription regulators in lipid synthesis, as observed for non-ruminants [32]. For instance, the increase in expression of *PPARG* in bovine mammary tissue during lactation indicated that milk fat synthesis might be under partial control by PPARγ [13]. The PPAR isotypes (α, δ, γ) are activated to various extents by long-chain fatty acids (LCFA) in MEC, hence, making them potential targets for fine-tuning metabolism via nutrients [33]. Kadegowda et al. [19] demonstrated that genes related to de novo FA synthesis (*ACACA, FASN*), TAG synthesis (*AGPAT6, DGAT1, LPIN1*), and transcriptional regulation of lipid synthesis (*SREBF1, INSIG1*) were upregulated in Mac-T cells by treatment with a PPARG agonist (rosiglitazone). Despite the lack of effect of mTOR on *PPARG,* the up-regulation of lipogenic genes in OPAA compared with OPAARMC indicates that EAA could indirectly alter PPARG, hence, play a role in de novo FA and TAG synthesis regulation [33].

In a similar manner, Blanchard et al. [34] working with adipocytes uncovered evidence of mTOR regulation of PPARG transcriptional activity in the absence of changes in *PPARG* mRNA expression. The authors suggested that the regulatory mechanisms might involve covalent and allosteric modulation of PPARG and/or its co-regulators [34]. In fact, the PPARG co-regulators *PPARGC1A* and *LPIN1* are substantially up-regulated during lactation in bovine mammary cells, indicating an important role in milk fat synthesis [13]. In that context, the upregulation of *LPIN1* in the rapamycin-free medium is noteworthy.

The up-regulation of *PPARD* was associated with greater expression of genes related to de novo FA synthesis (*ACACA, FASN*), desaturation (*SCD*), and intracellular FA transport (*FABP3*), all of which agrees with a positive correlation reported among these genes in a previous Mac-T cell study [35]. Further research to determine a role for *PPARD* in de novo FA synthesis will have to be performed because correlations were weaker compared with that for *SREBF1* [35].

Although we did not include a treatment devoid of EAA and insulin, the observed expression level of the lipogenic genes (*ACACA*, *FASN*, *SCD*) in response to OPAA provides some indication of EAA as regulators of transcription in bovine mammary cells. The insulin effect alone or in combination with a mixture of EAA on lipogenic gene expression (e.g. *FASN*, *SREBF1*) in cells such as hepatocytes is clear [11, 36]. In fact, the potent effect of EAA plus insulin in up-regulating *FASN* and *SREBF1* appears to require mTOR because incubating EAA plus insulin plus RMC completely prevented the up-regulation of lipogenic genes [36]. Data from hepatocyte incubations with Leu, Met, and Lys further confirmed that the presence of insulin is essential for up-regulation of *FASN* and other lipogenic genes [13]. Because lipogenesis is controlled differently in ruminants than non-ruminants [37], e.g. ruminant mammary tissue does not have an absolute requirement for insulin to perform lipogenesis, the lipogenic role of insulin signaling in the bovine mammary gland (via IRS1 and AKT1), and that of mTOR, might be more biologically-meaningful as lactation progresses [38], i.e. when sensitivity of the mammary gland to insulin is greater. A greater sensitivity of the mammary gland to insulin after peak lactation (i.e. when the cow reaches positive energy balance) would coincide with the greater expression of genes associated with insulin signaling in mammary tissue [38].

### Regulation of mammary lipogenic genes by the specific ratios of EAA

#### Lys to Thr ratios

Previous work with murine mammary cells revealed that an increase in Thr has a negative effect on mTOR and S6K1 phosphorylation status [9]. Conversely, Arriola-Apelo et al. [7] observed stimulatory effects of Thr on mTOR phosphorylation when incubated alone with mammary tissue slices. However, Thr is antagonistic to Ile and reduced the positive effect of the latter on phosphorylation status of mTOR and S6 [7]. The fact that both LT2.1 and LT1.3 (rapamycin-free) up-regulated *ACSS2, FABP3, ACACA, FASN, SCD, LPIN1, INSIG1, SREBF1*, and *PPARD* relative to OPAARMC indicates that a functional bovine mammary mTOR pathway is required for upregulation of lipogenic genes.

#### Lys to His ratio

Although LH3.0 resulted in up-regulation of several lipogenic genes, the fact that expression of *ACSS2*, *FASN*, *SREBF1*, and *INSIG1* was different compared with treatments like LT1.3 and LV1.6 also is indicative of unique effects of His on lipogenic gene transcription. These responses are indicative of a potentially novel and important role in regulating lipogenic gene transcription. Previous work with murine [9] mammary cells indicated that His is a potential inhibitor of mTOR, but our data and that from other groups [4–6] do not indicate a similar effect in bovine mammary cells. Although no direct data linking His with lipogenic gene transcription exist, at least in rat liver and muscle, it is clear that AA composition of dietary protein not only can modulate lipogenic enzyme activity but also gene transcription [39].

#### Lys to Val ratio

Among all treatments studied, the most significant effects on transcription compared with OAARMC were detected with LV1.6 (Fig. 1), e.g. greatest expression of *ACSL1*, *SREBF1*, *RXRA*, and *NR1H3*. In addition, it resulted in the lowest expression of *PPARG*. These data seem to indicate that lipogenic gene transcription is particularly sensitive to cellular availability of Val and that, below a certain level, it could have a negative effect. The fact that LV1.6 up-regulated *ACSL1* compared with other treatments is noteworthy because the expression of this gene at the onset of lactation in bovine mammary tissue is markedly up-regulated [13]. The down-regulation of *PPARG* expression with LV1.6 could be partly related with the up-regulation of *SREBF1*, *N1HR3*, and *RXRA*, i.e. a compensatory mechanism to help maintain lipogenic gene transcription.

### Regulation of mammary miRNA by EAA

The process of milk fat synthesis in mammary epithelial cells involves not only de novo synthesis but also TAG synthesis and fat droplet formation, all of which are potentially regulated by miRNA [40]. MicroRNA are short, non-coding molecules that have an important role in gene expression. Several studies have demonstrated that miRNA silences target mRNA by degradation or translation repression [15]. However, miRNA can also function to induce gene expression by targeting promoter sequences [41] or even increase protein translation by binding to complementary promoter sequences [42]. Although the biological functions of most miRNA are unknown, it is estimated that >30 % of protein-coding genes are regulated by miRNA [43].

The pattern of response of MIR27AB observed in this study is in agreement with previous reports demonstrating a negative correlation among MIR27 and genes related to fatty acid and TAG synthesis in goat mammary gland [44] and murine cells [14]. The mechanism responsible for such effect is through MIR27 directly targeting the 3’ untranslated region (UTR) of *PPARG* mRNA, decreasing its abundance [14], which does not agree with our *PPARG* results. It was expected that *PPARG* mRNA expression would be up-regulated because of the down-regulation of MIR27AB, but this was not observed.

Another miRNA considered anti-adipogenic is MIR130. MicroRNA 130 also targets *PPARG*, strongly repressing its expression by targeting two functional sites in the mRNA: the 3’ UTR and the coding region [45]. A recent study reported abundant expression of MIR130b in goat mammary gland tissue and revealed a negative adipogenic effect potentially through inhibition of *PPARGC1A* [46]. Similarly to what happens for *PPARG*, MIR130b potently repressed *PPARGC1A* expression by targeting both the *PPARGC1A* mRNA coding region and 3’UTR [46]. However, the lower expression of MIR130A in all rapamycin-free treatments does not agree with the decrease in *PPARG* expression. It might be possible that MIR130A does not function in the same manner as MIR130B.

The discrepancy between the response in expression of *PPARG* and the two *PPARG*-targeting miRNA indicates that regulation of both PPARG and other lipogenic genes is complex, involves multiple regulators, as well as post-transcription and post-translational regulation. In addition, PPARG itself regulates transcription of many miRNA. The lower expression of MIR27AB and MIR130A with OPAA relative to OPAARMC is noteworthy because it seems to indicate that mTOR somehow might be involved in regulating their expression.

In contrast to MIR27AB and MIR130A, MIR21 in human cells has a pro-lipogenic effect [47]. More specifically, MIR21 promotes an HBP1-mediated inhibition of p53 expression, which in turn, inhibits *SREBF1* expression [47, 48]. This relationship was confirmed in the treatments LT2.1, LT1.3, and LH3.0, in which both MIR21 and *SREBF1* as well as most of the lipogenic genes were up-regulated. Among all treatments, LV1.6 promoted the highest *SREBF1* expression, while MIR21 expression was lower compared with LT2.1, LT1.3 and LH3.0. The reason for these responses remains to be determined, as we are unaware of any known regulatory effect of EAA on the HBP1-p53-SREBP1c pathway [48]. Therefore, although without elucidating mechanisms of action, our results indicate important roles of MIR27AB, MIR130A and MIR21 in mammary lipogenesis, as well as in the relationship between EAA and lipid synthesis. Despite some of them having recognized regulatory functions in lipogenesis (MIR103, MIR378, MIR34A; [45], the other miRNA evaluated in this study were not affected by the treatments.

Most studies evaluating the roles of miRNA in regulation of lipid synthesis have been performed with murine or human cell lines. However, a recent publication identified several miRNA that change in expression in the mammary gland of cows after feeding a milk fat-depressing diet (MFD) [49]. Besides revealing novel miRNA expressed in bovine, that research revealed a total of 7 miRNA with a strong differential expression during MFD. Six of them (miR-199c, miR-199a-3p, miR-98, miR-378, miR-148b and miR-21-5p) were up-regulated, while 1 (miR-200a) was down-regulated. Therefore, our data add to the current knowledgebase and emphasizes the importance of miRNA in milk fat synthesis regulation in the context of EAA and the mTOR pathway.

## Conclusions

The observed up-regulation of lipogenic gene networks and the changes in expression of key miRNA involved in the control of lipogenic balance are indicative of a potentially important role of EAA ratios and mTOR signaling in the regulation of milk fat synthesis.

## Abbreviations

AA, amino acid; ACACA, Acetyl-coenzyme A carboxylase alpha; ACSL1, Acyl-CoA synthetase long-chain family member 1; ACSS2, Acyl-CoA synthetase short-chain family member 2; AGPAT6, 1-acylglycerol-3-phosphate O-acyltransferase 6; DGAT1, diacylglycerol acyltransferase 1; EAA, essential amino acids; EIF4E, eukaryotic translation initiation factor 4E; EIF4EBP1, eukaryotic translation initiation factor 4E-binding protein 1; FA, fatty acid; FABP3, fatty acid-binding protein, heart; FASN, fatty acid synthase; GADPH, glyceraldehyde 3-phosphate dehydrogenase; INSIG1, insulin induced gene 1; LCFA, long-chain fatty acids; LPIN1, lipin 1; Mac-T, bovine mammary epithelial cells; MEC, mammary epithelial cells; MFD, milk fat-depressing diet; MIR103, microRNA 103; MIR130a, microRNA 130a; MIR21, microRNA 21; MIR27ab, microRNA 27ab; MIR34a, microRNA 34a; MIR378, microRNA 378; MIR448, microRNA 448; miRNA, microRNA; MTOR, mechanistic target of rapamycin (Ser/Thr kinase); mTORC1, mTOR complex 1; NR1H3, liver X receptor α; PPARD, peroxisome proliferator-activated receptor beta; PPARG, peroxisome proliferator-activated receptor gamma; RHEB, ras homolog enriched in brain; RIN, RNA integrity; RMC, rapamycin; RPS6KB1, ribosomal protein S6 kinase beta-1; RPS9, ribosomal protein S9; RXRA, retinoid X receptor, alpha; SCD, stearoyl-CoA desaturase; SREBF1, sterol regulatory element-binding transcription factor 1; TAG, triacylglycerol; TSC1, tuberous sclerosis 1; UXT, ubiquitously expressed prefoldin like chaperone

## Additional file

Additional file 1: Table S1. GenBank accession number, hybridization position, sequence, amplicon size of primers used^1^. (DOCX 18 kb)
