# Supplementary material for: Essential amino acid ratios and mTOR affect lipogenic gene networks and miRNA expression in bovine mammary epithelial cells
Source: J Anim Sci Biotechnol. 2016 Aug 3;7:44. doi: 10.1186/s40104-016-0104-x (PMC4973084; doi:10.1186/s40104-016-0104-x)
Supplement: Additional file 1: Table S1. — GenBank accession number, hybridization position, sequence, amplicon size of primers used1. (DOCX 18 kb) [file 40104_2016_104_MOESM1_ESM.docx]

Suppl. Table 1. GenBank accession number, hybridization position, sequence, amplicon size of primers used^1^

| Accession # | Gene | Primers^2^ | Primers (5’-3’) | Bp^3^ |
| --- | --- | --- | --- | --- |
| BC134532 | *ACSS2* | F. 1881 | GGCGAATGCCTCTACTGCTT | 100 |
|  |  | R. 1970 | GGCCAATCTTTTCTCTAATCTGCTT |  |
| BC119914 | *ACSL1* | F. 1929 | GTGGGCTCCTTTGAAGAACTGT | 120 |
|  |  | R. 2047 | ATAGATGCCTTTGACCTGTTCAAAT |  |
| DN518905 | *FABP3* | F. 458 | GAACTCGACTCCCAGCTTGAA | 102 |
|  |  | R. 559 | AAGCCTACCACAATCATCGAAG |  |
| AJ132890 | *ACACA* | F. 3709 | CATCTTGTCCGAAACGTCGAT | 101 |
|  |  | R. 3809 | CCCTTCGAACATACACCTCCA |  |
| CR552737 | *FASN* | F. 6383 | ACCTCGTGAAGGCTGTGACTCA | 92 |
|  |  | R. 6474 | TGAGTCGAGGCCAAGGTCTGAA |  |
| BC112700 | *SCD* | F. 974 | AAAGAAAAGGGTTCCACGCTAA | 80 |
|  |  | R. 1053 | GGTTTGTAGTACCTCCTCTGGAACA |  |
| DY208485 | *AGPAT6* | F. 171 | AAGCAAGTTGCCCATCCTCA | 101 |
|  |  | R. 271 | AAACTGTGGCTCCAATTTCGA |  |
| NM_174693 | *DGAT1* | F. 177 | CCACTGGGACCTGAGGTGTC | 101 |
|  |  | R. 277 | GCATCACCACACACCAATTCA |  |
| DV797268 | *LPIN1* | F. 147 | TGGCCACCAGAATAAAGCATG | 101 |
|  |  | R. 247 | GCTGACGCTGGACAACAGG |  |
| CX736793 | *INSIG1* | F. 82 | CATCGACAGTCACCTTGGAGA | 108 |
|  |  | R. 189 | TCCAGTTTAGCACTAGCGTGGT |  |
| CO885324 | *SREBF1* | F.168 | AGCCTAGGCAATAGTAGTAGAGAAG | 84 |
|  |  | R.251 | GGCCTTAGTCAATAGGAGCTAGT |  |
| Y12420 | *PPARG* | F. 1356 | GAGCCCAAGTTCGAGTTTGC | 100 |
|  |  | R. 1455 | GGCGGTCTCCACTGAGAATAAT |  |
| AF229357 | *PPARD* | F. 1077 | ACAGTGACCTGGCTCTCTTCATC | 82 |
|  |  | R. 1158 | GCCTCCACCTGAGACACGTT |  |
| XM_005216424 | *NR1H3* | F. 1443 | GACCGACTGATGTTCCCAAG | 171 |
|  |  | R. 1613 | ACAGAAGACACGGAGGAGGA |  |
| XM_881943 | *RXRA* | F. 435 | CGCTCCTCAGGCAAGCA | 121 |
|  |  | R. 555 | TGTCAATCAGGCAGTCCTTGTT |  |
| BC111272 | *EIF4E* | F. 551 | AGGGAGGGTATACAAGGAAAGGTT | 101 |
|  |  | R. 651 | TTTTAGTGGTGGAGCCGCTC |  |
| NM_001077893 | *EIF4EBP1* | F. 169 | GATCATCTATGACCGGAAGTTCCT | 111 |
|  |  | R. 279 | TCATCGCCTGTAGGGCTAGTG |  |
| XM_002694043 | *MTOR* | F. 3452 | CCCCGATCGTGAAGTTATTTG | 141 |
|  |  | R. 3592 | GTGTGCGTACAATCGGATGAA |  |
| NM_001031764 | *RHEB* | F. 53 | GCTAAGATGCCGCAGTCCA | 75 |
|  |  | R. 127 | CGTCAACGAGGATTTCCCC |  |
| NM_205816 | *RPS6KB1* | F. 1286 | AACCAAAAATCCGATCCCCT | 101 |
|  |  | R. 1386 | TGAAGCACCTCTTCCCCAGA |  |
| BT021605 | *TSC1* | F. 452 | TACTGGGCCACGTCGTGAG | 102 |
|  |  | R. 553 | CGTCGGTGTCCATCTTGAGAC |  |

^1^*ACSS2* = Acyl-CoA synthetase short-chain family member 2; *ACSL1* = Acyl-CoA synthetase long-chain family member 1; *FABP3* = Fatty acid-binding protein, heart; *ACACA* = Acetyl-coenzyme A carboxylase alpha; *FASN* = Fatty acid synthase; *SCD* = Stearoyl-CoA desaturase; *AGPAT6* = 1-acylglycerol-3-phosphate O-acyltransferase 6; *DGAT1* = Diacylglycerol acyltransferase 1; *LPIN1* = Lipin 1; *INSIG1* = Insulin induced gene 1; *SREBF1* = Sterol regulatory element-binding transcription factor 1; *PPARG* = Peroxisome proliferator-activated receptor gamma; *PPARD* = Peroxisome proliferator-activated receptor beta; *NR1H3* = Liver X receptor α; *RXRA* = retinoid X receptor, alpha; *EIF4E* = Eukaryotic translation initiation factor 4E; *EIF4EBP1* = Eukaryotic translation initiation factor 4E-binding protein 1; *MTOR* = Mechanistic target of rapamycin (Ser/Thr kinase); RHEB = Ras homolog enriched in brain; *RPS6KB1*= Ribosomal protein S6 kinase beta-1; *TSC1* = Tuberous sclerosis 1.

^2^Primer direction (F - forward; R - reverse) and hybridization position on the sequence

^3^Amplicon size in base pair (bp)
